# Vaccine Adjuvants and Delivery Systems: A Comprehensive Review

**DOI:** 10.3390/ijms27104271

**Published:** 2026-05-11

**Authors:** Alexis Hipólito García, Juan Bautista De Sanctis

**Affiliations:** 1 Institute of Immunology “Nicolás Enrique Bianco”, Faculty of Medicine, Universidad Central de Venezuela, Caracas 1050, Venezuela; 2 Institute of Molecular and Translational Medicine, Faculty of Medicine and Dentistry, Palacky University, Hněvotínská 1333/5, 779 00 Olomouc, Czech Republic; 3 Czech Advanced Technology and Research Institute, Palacky University in Olomouc, Hnevotinska 1333/5, 779 00 Olomouc, Czech Republic

**Keywords:** vaccines, adjuvants, nanoformulations, delivery systems, aluminum salts, lipid nanoparticles, oil emulsions

## Abstract

Adjuvants play a crucial role in increasing vaccination efficacy. While aluminum salts have historically been the most common adjuvants, recent research has turned to new compounds with enhanced adjuvant properties and improved safety. Cutting-edge nanotechnology, leveraging nanoformulations and novel delivery systems, has enhanced efficacy while reducing adverse effects. Microparticles, emulsions, and immunostimulants are now essential tools due to their significant potential for vaccine production. Additionally, advanced drug delivery systems (DDSs) have been developed using sophisticated technologies to expedite and optimize drug and vaccine delivery to specific target sites, thereby maximizing therapeutic efficacy and minimizing systemic accumulation. The latest DDSs offer numerous advantages over conventional drug delivery systems, including heightened performance, precision, and efficiency. These DDSs, comprising nanomaterials or miniaturized devices, feature multifunctional components that are biocompatible and biodegradable, with high viscoelasticity, thereby extending their circulating half-life. This review aims to provide an in-depth and up-to-date overview of adjuvants and technological advancements in vaccine delivery systems.

## 1. Introduction

**Figure 1:**
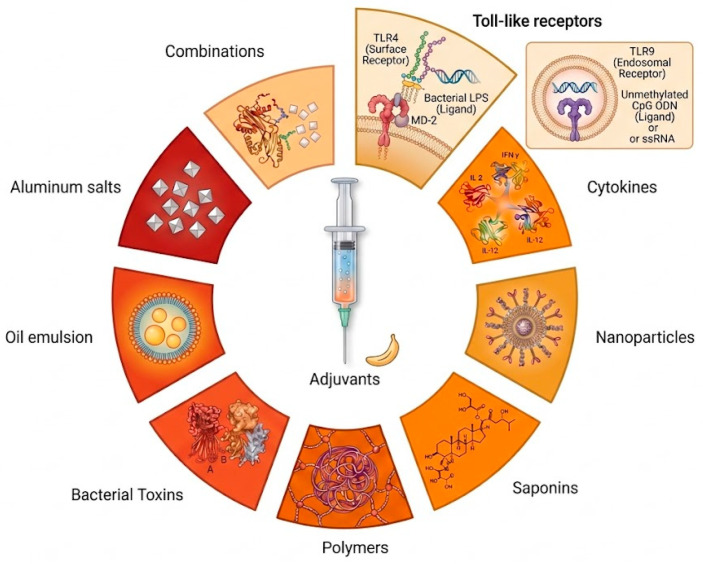
Schematic representation of the diverse landscape of vaccine adjuvants and their molecular targets. The illustration highlights fundamental categories such as aluminum salts, oil-in-water emulsions, and saponins, which act through distinct mechanisms to enhance immunogenicity. Furthermore, it incorporates advanced delivery platforms such as polymers and nanoparticles, alongside potent biological modifiers, including cytokines and bacterial toxins. As part of this comprehensive repertoire, specific Toll-like receptor (TLR) ligands—such as TLR4 and TLR9 agonists—are depicted to show their role in triggering innate signaling pathways. These distinct agents are employed alone or in strategic combinations to refine the overall magnitude, duration, and specificity of the resulting adaptive immune response. The image was generated using Gemini 3 Pro Image (Nano Banana, https://ai.google.dev/gemini-api/docs/models/gemini-3-pro-image, access is available from 1 March to 30 April 2026).

Adjuvants play a crucial role in modern immunotherapy and vaccine development by enhancing immune responses to prophylactic vaccines. They improve antigen recognition and activate key immune cells, fostering adaptive immunity. Various classes of adjuvants exist, ranging from traditional mineral salts to advanced agents such as Toll-like receptor agonists and nanoparticles (Figure 1). However, the emergence of advanced immunotherapies has revealed the limitations of traditional adjuvants, particularly their narrow spectrum of immune activation. For instance, many conventional adjuvants often fall short of effectively polarizing Th1 or Th17 immune responses, which are indispensable for combating intracellular pathogens and tumors, or of inducing robust cross-presentation, which is necessary for cytotoxic T lymphocyte (CTL)-mediated immunity [1,2,3]. Figure 1 represents the adjuvants that have been researched and developed. These components have been evaluated at multiple levels over time, and the majority are currently used in several vaccine formulations.

The evolution in adjuvant development marks a shift from empirical discovery to a strategic design approach. Historically, adjuvants were included by chance, with little understanding of their biological effects. However, improved understanding of innate immune mechanisms and pattern recognition receptor (PRR) signaling has led to a more targeted approach to immunomodulation. This paradigm shift enables fine-tuning of immune responses, promoting Th1 responses for cellular immunity or Th2 responses for humoral immunity. This precision enhances vaccine efficacy, minimizes off-target effects, and improves the safety profiles of vaccine formulations [4].

Emerging agents, such as the synthetic TLR3 ligand ARNAX, synthetic DNA-capped double-stranded RNA (dsRNA) molecule, CpG ODNs, STING agonists, β-glucan derivatives, and synthetic poly(I:C), employ distinct mechanisms to modulate the immune response. Adjuvants not only enhance immune responses but also reduce the antigen doses required, thereby improving vaccine cost-effectiveness and accessibility [4,5]. Modern adjuvants are no longer categorized merely as agents that increase the magnitude of an immune response. Instead, they are viewed as biological switches capable of polarizing T-helper responses toward Th1 (cellular), Th2 (humoral), or Th17 (mucosal) phenotypes depending on the specific requirement of the pathogen or malignancy being targeted. This precision is achieved through the targeted engagement of PRRs, the innate immune system’s primary sensors [6]. The review aims to provide an overview of adjuvants and delivery systems, offering insight into recent developments in this area.

## 2. Methodology

A comprehensive literature search was conducted across electronic databases, including PubMed/MEDLINE, Scopus, and Web of Science, for peer-reviewed articles published through April 2026. The search strategy focused on identifying high-quality evidence regarding vaccine adjuvants and delivery systems with a specific emphasis on their application in human clinical settings. Key search terms and descriptors utilized included “vaccines,” “adjuvants,” “nanoformulations,” “delivery systems,” “aluminum salts,” “lipid nanoparticles,” and “oil emulsions”.

The selection criteria prioritized human-centered research and translational immunology, specifically targeting the clinical efficacy and safety profiles of licensed adjuvant systems, including AS01, AS03, AS04, and Matrix-M™. Particular attention was given to studies describing the molecular mechanisms of human PRRs engagement and the cytokine-driven T-helper cell differentiation required for robust immunity. Evidence was further gathered regarding the impact of diverse biological variables—including age, genetic variation, biological sex, and comorbidities—on the human immune response to various vaccine platforms.

Finally, the search incorporated the latest clinical advancements in advanced drug delivery systems (DDSs), with a primary focus on mRNA-lipid nanoparticle (LNP) formulations and the emerging role of exosomes as potent biological adjuvants. Strategic focus was placed on technological milestones through early 2026, including needle-free administration platforms, thermostable microneedle patches, and the integration of artificial intelligence frameworks to predict T-cell recognition with high molecular accuracy. Only English-language publications involving human subjects or direct clinical applications were synthesized to ensure the highest level of evidence for this review.

## 3. Carriers, Adjuvants, Immunostimulants, Small Molecules, and Combination Adjuvants

### 3.1. Carriers

Carrier proteins function as molecular transporters covalently conjugated to the antigen, a linkage essential for polysaccharides with low intrinsic immunogenicity [7]. Pure polysaccharides were historically classified as T-cell-independent antigens because they couldn’t be processed by T lymphocytes, thereby preventing B lymphocytes from undergoing isotype switching or creating immunological memory. Conjugating these polysaccharides to carrier proteins, such as CRM197 or tetanus toxoid, enables B lymphocytes to recognize and internalize the entire conjugate [7,8,9].

Once internalized, the protein is degraded into peptides and presented via the MHC-II pathway to helper T cells, transforming the immune response into a T-cell-dependent process. This interaction stimulates germinal center formation, leading to the maturation of B cells into high-affinity memory cells and long-lived plasma cells. This mechanism is essential to the effectiveness of modern vaccines, such as the 13-, 15-, and 20-valent pneumococcal conjugate vaccine [10]. Carrier proteins are essential for attracting T lymphocytes, but they usually lack intrinsic adjuvant properties and are often combined with mineral salts to enhance immune response.

The evolution of conjugate vaccines is moving towards higher-valency formulations and site-specific bioconjugation to improve structural consistency. Future developments are exploring recombinant or synthetic carrier proteins to reduce carrier-induced epitope suppression and enhance T lymphocyte recruitment. Additionally, new adjuvant systems are being integrated to widen the protective window and ensure stronger, longer-lasting immunity in diverse populations [9,10].

### 3.2. Adjuvants

Vaccine adjuvants are mainly categorized into two types: immunostimulants and delivery systems. Many adjuvants combine these properties, highlighting the complex interactions within the immune system [11]. Immunostimulants activate antigen-presenting cells (APCs) by engaging PRRs, which are crucial for APCs’ maturation and activation [12,13]. In contrast, delivery systems primarily facilitate the uptake, transport, and presentation of antigens by APCs, ensuring prolonged immune exposure and, consequently, high, sustained antibody levels [14,15].

**Figure 2:**
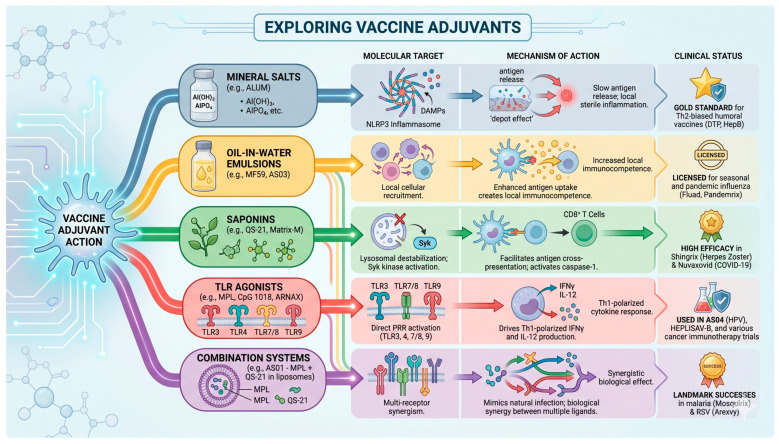
Exploring the landscape of vaccine adjuvants reveals five primary categories, starting with mineral salts like Alum, which target the NLRP3 inflammasome to promote slow antigen release in Th2-biased vaccines. Oil-in-water emulsions, such as MF59 and AS03, enhance local cellular recruitment and antigen uptake, thereby boosting immunocompetence in seasonal and pandemic influenza vaccines. Saponins, including QS-21 and Matrix-M, destabilize lysosomes and activate Syk kinase, facilitating CD8+ T cell cross-presentation for high-efficacy vaccines such as Shingrix and Nuvaxovid. TLR agonists, such as monophosphoryl lipid A (MPL), directly activate pattern recognition receptors to drive Th1-polarized cytokine responses in HPV- and cancer-immunotherapy trials. Ultimately, combination systems like AS01 leverage multi-receptor synergism to mimic natural infection, achieving landmark success in vaccines against malaria and RSV. The image was generated using Gemini AI.

Immunostimulants activate the immune system by signaling danger from pathogens or damaged cells through molecules recognized by PAMPs and DAMPs. PAMPs include lipopolysaccharide (LPS), bacterial DNA, liposomes, glycolipids, flagellin, and viral components, including glycoproteins and nucleic acids (dsRNA, ssRNA, 5-triphosphate RNA) [15]. Heat shock proteins (HSPA1A and HSPB1), High Mobility Group Box 1 (HMGB1), histones, mitochondrial components, and uric acid interact with DAMP receptors [16,17,18]. Figure 2 provides a comprehensive schematic representation synthesizing the complex landscape of the diverse adjuvant classes currently employed in modern immunology. It offers an integrated summary that systematically links the chemical identities of these agents to their specific molecular targets, such as PRRs, while detailing the underlying mechanisms of action that facilitate modulation and enhancement of the adaptive immune response.

### 3.3. Mechanisms of PRRS Engagement by Immunostimulants

Binding PAMPs, DAMPs, or their mimics to PRRs triggers conformational changes that initiate intracellular signaling cascades involving adaptor molecules such as MyD88 and TRIF. This activates transcription factors, such as NF-κB, MAPKs, and IRFs, leading to the production of proinflammatory cytokines and chemokines that are vital for the host response to infection and adaptive immunity [19,20]. Importantly, PRR signaling is context-dependent, enabling the immune system to tailor its response to the type and location of the threat. The engagement of diverse ligands triggers distinct intracellular signaling pathways, resulting in specific cytokine profiles that orchestrate T helper cell differentiation (e.g., Th1, Th2, Th17) and CTL generation. This mechanistic insight enables the strategic design of adjuvants to elicit vaccine responses tailored to the requirements of specific pathogens [20,21,22,23,24].

The localization of PRRS receptors is crucial for activating immune responses. PRRs are located on cell surfaces, within endosomes, and in the cytosol, allowing the immune system to detect hazards effectively [25,26]. Nucleic acid-sensing PRRs in endosomes or the cytosol detect intracellular viral replication, activating pathways such as TLR3 and RLRs, which lead to Type I interferon production and enhance antiviral immunity. Additionally, the distinct responses of TLR4 to MPL and LPS highlight how different stimuli engage distinct signaling pathways. Understanding PRR signaling mechanisms is essential for developing effective adjuvants [25,26].

### 3.4. Antigen-Presenting Cells (APCs) Maturation and Activation by Immunostimulants

Understanding PRR signaling is crucial for developing effective adjuvants. During maturation, APCs enhance antigen processing, forming MHC-peptide complexes that serve as Signal 1 for T lymphocyte activation. These complexes are presented on APCs, predominantly by dendritic cells. Mature APCs also express higher levels of co-stimulatory molecules, such as CD40, CD80 (B7.1), and CD86 (B7.2), which are vital for activating naive T lymphocytes. Additionally, co-stimulators such as CD54 (ICAM-1) and the 4-1BB ligand play essential roles, with CD86 being notably abundant on DCs and crucial for the formation of the immunological synapse with T lymphocytes [27,28].

### 3.5. Role of Cytokine Secretion in APCs Activation

Activation of PRRs triggers the secretion of inflammatory cytokines and chemokines, known as Signal 3 in T cell activation, which is essential for the adaptive immune response. Activated APCs produce: (1) pro-inflammatory cytokines (e.g., IL-1, IL-6, IL-12, IL-23, TNF-α); (2) T helper-polarizing cytokines (e.g., IL-12, IL-23, IL-15, type I interferons); (3) immunosuppressive cytokines (e.g., IL-10, TGF-β); and (4) chemokines that guide DCS migration. Notably, CCL19 and CCL21 lead DCS to lymph nodes via CCR7, while CXCL12 and CXCL14 direct them to inflammation sites. CCL22 and CCL17 also influence DCS positioning within tissues [29].

### 3.6. DCs: The Master Orchestrators of Adaptive Immunity

**Figure 3:**
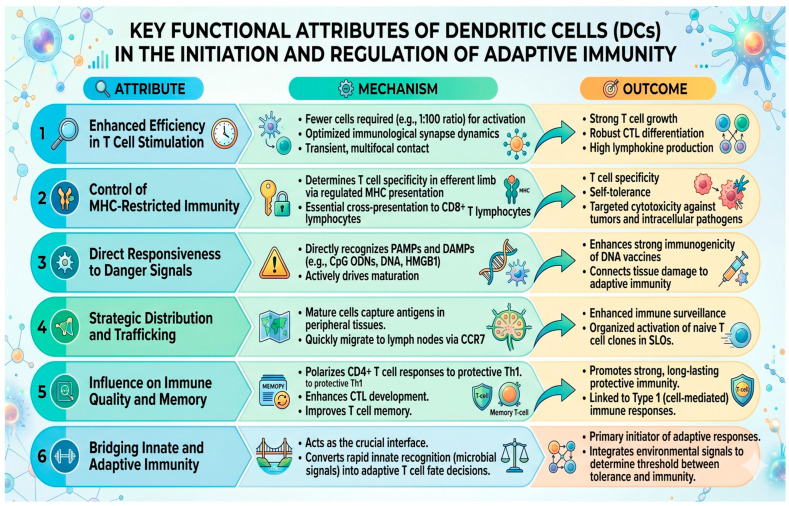
Key functional attributes of dendritic cells (DCs) highlight their central role in initiating and regulating adaptive immunity, starting with their remarkable efficiency in T cell stimulation through optimized synapse dynamics that lead to robust cytotoxic T lymphocyte (CTL) differentiation. They maintain control over MHC-restricted immunity by facilitating essential cross-presentation to CD8+ T lymphocytes, ensuring both targeted cytotoxicity and self-tolerance. By directly responding to danger signals like PAMPs and DAMPs, DCs trigger maturation and effectively connect localized tissue damage to systemic adaptive responses. Their strategic distribution and CCR7-mediated trafficking allow for efficient antigen capture in the periphery and organized T cell activation in secondary lymphoid organs. Additionally, DCs shape the quality of the immune response and long-term memory by polarizing CD4+ cells toward a protective Th1 response. Serving as the primary bridge between innate and adaptive systems, these cells integrate environmental signals to determine the vital threshold between immune tolerance and active defense. The image was generated using Gemini AI.

DCs are considered nature’s adjuvants because they initiate and shape adaptive immune responses. They are effective in capturing, processing, and presenting antigens, supporting the expansion of Th1 helper T lymphocytes and cytotoxic T lymphocytes [30]. Antigen-bearing DCs can elicit strong antimicrobial and anti-tumor immunity even without extra adjuvants [30,31,32]. Several unique attributes make DCs superior APCs and prime targets for immunostimulant action. They are summarized in Figure 3. Key elements that enhance the efficacy of vaccine responses include effective stimulation of T cells, regulation of MHC presentation, responsiveness to danger signals, the strategic distribution and migration of dendritic cells, contributions to sustained and robust T cell memory responses, and the interaction between innate and adaptive immune responses. In conclusion, the role of dendritic cells in facilitating a strong, specific response to an antigen is essential to the overall effectiveness of vaccines.

### 3.7. The Critical Role of DCs Maturation as a Control Point for Initiating Immunity

Vaccine antigens are most effective when presented by mature DCs, which stimulate T lymphocytes, which are essential for a robust adaptive immune response [31]. Microbial products, such as LPS, double-stranded RNA, and CpG-ODN, along with cytokines like GM-CSF, TNF-α, IL-1β, lipid products like PGE2, and TNF family members like CD40L, effectively stimulate dendritic cell maturation both in vitro and in vivo [30,31,32,33]. This maturation requires upregulation of CCR7 receptors to enable migration to lymph nodes, a critical step in T cell priming [34]. Conversely, inadequate DC maturation triggered by DNA vaccines or delivery strategies can lead to tolerance and immune suppression [32,33]. Immature DCs may promote tolerogenic cross-presentation, inducing regulatory T lymphocytes or deletion tolerance, which prevents the immune system from responding to self-antigens [35].

Adjuvants serve as critical regulators of the DC migratory journey, moving beyond simple immune stimulation to precisely calibrate the cellular mechanics required for adaptive immunity. As Olson et al. [36] detail, this process is governed by a triad of Rho-family GTPases-Rac1, Cdc42, and RhoA, which manage the actin polymerization and nuclear protection necessary for DCs to navigate the dense extracellular matrix and enter the lymphatic vasculature. By modulating these biomechanical pathways and the expression of navigation receptors such as CCR7, modern adjuvants ensure that antigen-carrying sentinels maintain directional persistence to reach secondary lymphoid organs effectively. Furthermore, targeted interventions can correct the “migratory bottlenecks” that frequently lead to vaccine failure in aged or genetically predisposed populations. Research by Zhao et al. [37] highlights a decisive signaling checkpoint in which hyperactivation of p38α suppresses ERK-dependent “go” signals required for DC movement. This defect can be bypassed through innovative strategies, such as oral yeast-derived nanoparticles (YNPs), which engage the gut-immune axis to upregulate CCR7 and restore DC trafficking to distant lymph nodes. Such precision adjuvancy “unlocks” the migratory potential of otherwise stationary cells, offering a noninvasive pathway to reverse immunosenescence and enhance systemic vaccine efficacy [37].

## 4. Types of Adjuvants and Their Evolution over Time

**Figure 4:**
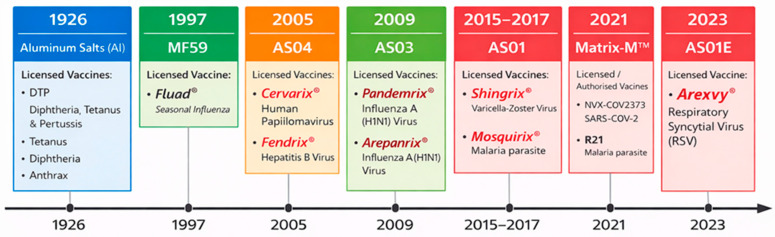
Timeline of licensed vaccine adjuvants (1926–2023). Aluminum salts (1926) were the first widely used adjuvants. Subsequent milestones include MF59 (1997), AS04 (2005), AS03 (2009), AS01 (2015–2017), Matrix-M™ (2021), and AS01E (2023), reflecting the progressive evolution of modern vaccine adjuvant platforms. The image was generated using Gemini AI.

The evolution of adjuvant technology reflects a paradigm shift toward precision immunology, where the focus has moved beyond simple immune stimulation to the fine-tuning of specific cellular responses. Modern research and development efforts now integrate a wide spectrum of chemical and biological platforms, including novel combinations designed to optimize vaccine efficacy and safety. An overview of these prominent adjuvant classes and their structural diversity is illustrated in Figure 4.

### 4.1. Aluminum Salt Adjuvants

While traditional aluminum salts have been the gold standard for nearly a century [38], their inability to induce Th1-mediated cellular immunity has led to the development of next-generation alternatives. Layered Double Hydroxides (LDHs), reengineered from first-line antacid drugs, have emerged as a potent class of “NanoAlum” that overcomes these historical limitations [39,40].

NanoAlum offers several transformative advantages over conventional salts: Balanced Immunogenicity: Unlike traditional alum, which is strictly Th2-biased, LDH nanoparticles can be engineered to induce both robust antibody responses and Th1-polarized CD8+ cytotoxic T lymphocyte (CTL) immunity [39,40]. This is achieved by tuning the metal cation ratios (e.g., Mg/Al) or by doping the layers with cations such as Zn^2+^, Cu^2+^, or Fe^3+^, which can activate the NF-κB and NLRP3 inflammasome pathways [39,40]. Tumor Microenvironment (TME) Modulation: A critical breakthrough identified by Su et al. [40] is LDH’s ability to act as a pH-responsive neutralizer. In solid tumors, NanoAlum persistently neutralizes TME acidity, converting an immunosuppressive environment (characterized by M2-like macrophages and Tregs) into an immunoregulatory one that favors tumor-killing M1-like macrophages and CD8+ T cells. Rapid Endosomal Escape: LDH nanoparticles are readily internalized and exhibit an intrinsic capacity to escape from endosomes (typically within 30 min). This allows the antigen payload to reach the cytosol directly, facilitating efficient cross-presentation on MHC-I molecules, a process that traditional alum aggregates (1–20 μm) cannot efficiently perform [40].

### 4.2. Oil-in-Water Emulsion Adjuvants

#### 4.2.1. MF59

Oil-in-water (O/W) emulsions, such as MF59 and AS03, disperse microscopic squalene droplets in an aqueous phase, stabilized by surfactants, such as Polysorbate 80, which reduce interfacial tension and prevent droplet coalescence [41]. MF59 contains 4.3% squalene and 0.5% each of Tween 80 and Span 85 in a citric acid buffer. This biodegradable formulation, with an average droplet size of 160 nm, was originally designed for antigen delivery but later demonstrated strong adjuvant properties [41,42,43].

This mechanism enhances antigen uptake by antigen-presenting cells and stimulates innate immune signaling at the injection site. It is crucial for improving antibody response, as demonstrated by the Fluad^®^ vaccine, which uses MF59 for better protection in the senior population, and pandemic vaccines like Pandemrix™ and Arepanrix™, which use AS03 for dose-sparing effects. The evolution of these emulsions involves integrating synthetic TLR4 agonists to further polarize the immune response, ensuring that next-generation vaccines can provide robust, cross-reactive immunity against emerging viral threats and highly mutable pathogens [43,44].

The MF59-adjuvanted influenza vaccine was licensed in 1997 for the senior population and is approved in over 30 countries, including the U.S. Nearly 100 million doses of MF59-adjuvanted H1N1 pandemic vaccines have been given to groups such as pregnant women and young children. MF59 boosts vaccine effectiveness by increasing antibody levels and enhancing long-term memory T and B lymphocytes. Its efficacy has been demonstrated in diverse populations, including children and older adults [45]. Ongoing studies are exploring its application to other vaccine antigens and platforms, as well as its mechanisms of action.

#### 4.2.2. AS03

AS03 is an oil-in-water emulsion adjuvant (squalene, DL-tocopherol, and polysorbate 80) for pandemic influenza vaccines, enhancing antibody and CD4+ T cell responses in recombinant COVID-19 vaccine trials and providing better protection than non-adjuvanted vaccines [46,47]. Licensed for use with pandemic influenza vaccines, it has been evaluated in trials of recombinant COVID-19 vaccines. AS03 significantly enhances antibody and CD4+ T cell responses, providing superior protection compared to non-adjuvanted vaccines. Further research indicates that AS03 can temporarily downregulate genes involved in lipid metabolism in lymph nodes. These genes are linked to changes in lipid composition and the unfolded protein response. In humans, elevated IL-6 and IP-10 (CXCL10) levels within 24 h post-vaccination are associated with stronger antibody responses. AS03 and MF59 are squalene-based adjuvants that likely share common activation pathways, although the exact roles of pathways such as IRE1α and RIPK3 remain to be clarified [48]. The future may see specialized adjuvants with carefully chosen components to achieve targeted outcomes, such as strong humoral, cellular, or mucosal immunity. These adjuvants can also be tailored to specific populations, including older adults and individuals with compromised immune systems [49].

#### 4.2.3. AS04

The development of AS04 and AS01 highlights the effectiveness of multi-component adjuvant systems. AS04, when combined with MPL, a TLR4 agonist, and aluminum salts, has shown better outcomes than alum alone; AS01 enhances this by incorporating MPL and QS-21 into a liposomal delivery system, where their synergy is crucial for its strong adjuvant effect [50,51]. This progression reflects a trend toward more sophisticated adjuvants that integrate multiple immunostimulants and delivery methods to achieve synergistic effects, addressing limitations such as QS-21′s toxicity. This approach marks a significant advancement in vaccine design, focusing on optimized immune responses, particularly in older populations.

To date, this adjuvant system has been successfully integrated into two primary clinical applications: the bivalent human papillomavirus (HPV) types 16 and 18 vaccine, marketed as Cervarix™ [52], and the specialized hepatitis B vaccine, Fendrix^®^ [53]. Fendrix^®^ is presently indicated for use in patients with renal insufficiency, such as those undergoing hemodialysis, to overcome the characteristic suboptimal immune signaling observed in this population.

#### 4.2.4. AS01

AS01 is used in several licensed vaccines. This adjuvant combines MPL, the TLR4 ligand in AS04, and QS-21, a purified glycoside from *Quillaja Saponaria* [54]. While QS-21 enhances immune responses, its use as a standalone has raised concerns about tolerability. AS01 addresses this by encapsulating MPL and QS-21 within liposomes containing cholesterol, thereby reducing QS-21′s reactogenicity. In the murine model, QS-21 activates caspase-1 in lymph node macrophages, but its adjuvant effect is independent of the NLRP3 pathway [55]. It is taken up via cholesterol-dependent endocytosis, leading to lysosomal destabilization and SYK kinase activation. This synergy is essential for AS01′s strong effect, enhancing poly-functional CD4+ T cell responses and inducing effective antibodies. This dual activation is significant for addressing immunosenescence in older populations. Further research is needed to fully understand the mechanisms of AS01 in humans.

The AS01 adjuvant system is currently incorporated into several breakthrough licensed vaccines, demonstrating a versatile capacity to enhance both humoral and cellular immunity. This includes Shingrix^®^ (Herpes Zoster [56], which achieves over 90% efficacy in adults aged 50 and older by significantly boosting T-cell memory; Mosquirix^®^ (Malaria) [57], the first vaccine to elicit high antibody titers and robust CD4+ T-cell responses in vulnerable pediatric populations; and Arexvy (RSVPreF3) [58], approved in 2023. Specifically designed for adults aged 60 and older, Arexvy combines a stabilized prefusion F protein with the AS01E adjuvant system to effectively bolster the immune response against severe lower respiratory tract disease (LRTD) caused by the respiratory syncytial virus.

Saponins are potent immunostimulants, and immunostimulatory complexes (ISCOMs) enhance vaccine delivery by combining antigens with cholesterol, phospholipids, and saponins to form 40-nm cage-like nanoparticles. Incorporating saponins into this lipid matrix is crucial, as it provides adjuvant properties and reduces the hemolytic toxicity of free saponins [59]. This structure encapsulates hydrophobic antigens for sustained release and improved uptake by antigen-presenting cells. Consequently, ISCOMs function as both high-efficiency carriers and immune activators, mimicking pathogen morphology to bridge innate and adaptive immunity by robustly recruiting dendritic cells and macrophages.

The clinical utility of this technology has been further expanded by ISCOMATRIX, a lipid-saponin framework that maintains the structural integrity of the complex without a pre-incorporated antigen. This allows for greater flexibility in vaccine formulation and precise dosage control during preparation. Both ISCOM and ISCOMATRIX have consistently outperformed traditional delivery systems in antigen presentation across a broad spectrum of targets, including HPV, HIV, and cancer immunotherapy. By using refined saponin fractions such as QS-21 and QuilA, these systems maintain a favorable safety profile while eliciting balanced cellular and humoral responses. Ultimately, ISCOMs exemplify rational vaccine design, providing a potent model for next-generation immunotherapies that achieve maximal efficacy with minimal antigenic and adjuvant requirements [60,61].

#### 4.2.5. CpG Oligodeoxynucleotides (ODNs)

ODNs mimic bacterial DNA and activate the immune system by binding to TLR9, which is expressed in various immune cells, including B cells, plasmacytoid dendritic cells, and macrophages [62]. This binding triggers the production of proinflammatory cytokines (IL-6, TNF-α, IL-12). It enhances the maturation of antigen-presenting cells, typically resulting in a Th1-biased immune response that helps combat intracellular pathogens or cancer [62].

The clinical use of free CpG ODNs is limited by rapid degradation, poor cellular uptake, and nonspecific distribution [63]. To address these issues, various nanomaterial-based delivery systems are being developed to enhance stability, targeting, and controlled release of CpG ODNs. Despite progress in enhancing immunostimulatory activity, challenges remain in elucidating the molecular mechanisms underlying nanocarrier effects, ensuring long-term safety and biocompatibility, and translating preclinical results into scalable, clinically relevant formulations. The integration of nanotechnology with immunotherapy, facilitated through diverse delivery platforms such as DNA nanostructures and lipid-based carriers, holds promise for advancing vaccines and therapeutics, particularly in cancer treatment and personalized medicine. By enabling protection and targeted delivery, nanotechnology transforms unstable compounds into viable therapeutic agents, extending its impact beyond CpG ODNs to other sensitive biomolecules [64,65].

In the contemporary global landscape, the following immunizations have been formally authorized for clinical distribution, integrating synthetic CpG oligodeoxynucleotide (ODN) motifs to bolster immune efficacy: HEPLISAV-B^®^ (Hepatitis B Vaccine [Recombinant], Adjuvanted), developed by Dynavax Technologies, was granted U.S. FDA approval in 2017 utilizing the CpG 1018^®^ adjuvant [66]. Subsequently, CORBEVAX™, a protein subunit-based COVID-19 vaccine, received its initial Emergency Use Authorization (EUA) in 2021, leveraging the CpG 1018^®^ platform to enhance immunogenicity [67]. Furthermore, in 2023, the FDA approved CYFENDUS^®^ (Anthrax Vaccine Adsorbed, Adjuvanted) for post-exposure prophylaxis; this formulation incorporates the CpG 7909 adjuvant to facilitate an accelerated and robust immune response against Bacillus anthracis [68].

#### 4.2.6. Matrix-M™

Matrix-M™ is a saponin-based adjuvant designed for modern subunit vaccines. It consists of two purified fractions of *Quillaja saponaria* saponins, combined with cholesterol and phospholipids to create ~40-nm nanoparticles. This defined blend (approximately 85% Matrix-A and 15% Matrix-C) maximizes immunostimulant potency while improving tolerability. Matrix-M exhibits vigorous adjuvant activity, allowing for significant antigen dose-sparing, and remains stable in solution at 2–8 °C for years [69].

Matrix-M saponins released from acidic lysosomes promote antigen escape into the cytosol for cross-presentation to CD8+ T lymphocytes via MHC class I. Intramuscular injection of Matrix-M–adjuvanted vaccines rapidly activates the innate immune system by recruiting neutrophils, monocytes, and antigen-presenting cells to the injection site. These cells transport the antigen to draining lymph nodes, improving antigen presentation. Matrix-M activates innate signaling pathways, such as the NLRP3 inflammasome, promoting the release of pro-inflammatory cytokines (IL-1β, IL-18) [70,71,72,73]. This enhances T-cell activation and Th1-type immunity, resulting in stronger adaptive responses, higher antibody titers, and robust memory B-cell development. Preclinical studies show that Matrix-M induces multifunctional CD4+ and CD8+ T lymphocytes and potent neutralizing antibodies, providing long-lasting immunity compared to unadjuvanted vaccines. Its dose-sparing effect also leads to enlarged, cell-rich lymph nodes, effectively priming adaptive responses even with low antigen doses.

Several immunological characteristics influence the clinical efficacy of vaccines, including age, genetic variation in HLA, biological sex, comorbidities, vaccine type, adjuvants, obesity, and nutrition, among others [74]. For instance, the NVX-CoV2373 SARS-CoV-2 spike nanoparticle vaccine (Novavax) requires the Matrix-M adjuvant to achieve 90–96% efficacy against symptomatic COVID-19 in Phase 3 trials [75,76]. Matrix-M enhances both humoral and cellular immunity, leading to higher antibody levels and stronger CD4+ T-cell responses. Furthermore, research conducted by Datoo et al. revealed that the formulation of the R21 malaria vaccine with the Matrix-M adjuvant induced a robust production of anti-CSP antibodies. This heightened immune response was associated with 74–77% protective efficacy against *Plasmodium falciparum* in African pediatric populations, further validating the vaccine’s pivotal role in reducing disease burden in endemic regions [77]. For seasonal influenza, Matrix-M adjuvanted vaccines elicited broader immunity than standard vaccines, with higher antibody responses and stronger multifunctional T-cell responses against drifted A(H3N2) strains [78]. Overall, Matrix-M significantly enhances immune response across diverse vaccine targets, including COVID-19, malaria, and influenza.

## 5. Adjuvant Combinations

Modern vaccinology has evolved from the empirical use of single substances toward the rational design of “Adjuvant Systems” (AS), which combine multiple immunostimulatory molecules to elicit a specific and potent immune profile [79,80]. While traditional aluminum salts (alum) have been the standard for decades, they are often insufficient for protecting against complex intracellular pathogens such as malaria or tuberculosis. By integrating multiple ligands targeting PRRs, these systems mimic the natural complexity of infections to activate multiple innate immune pathways simultaneously [81].

Several examples of successful adjuvant combinations are: (1) The AS01 system, TLR4 agonist monophosphoryl lipid A (MPL) with the saponin QS-21, in a liposomal carrier, which is effective in vaccines, described before, Shingrix and Mosquirix, helping counteract immunosenescence in older populations for sustained protection. (2) The AS04 system, which pairs MPL with aluminum salts, boosts Th1-biased immune responses in vaccines such as Cervarix™ (for HPV) and Fendrix^®^ (for hepatitis B), outperforming those containing aluminum alone. (3) Emulsion-based systems like AS03 boost antibody response in pandemic influenza vaccines, enabling lower doses. (4) The saponin-based Matrix-M adjuvant has demonstrated strong efficacy in the Novavax COVID-19 and R21 malaria vaccines for both children and adults. Advances in systems biology, such as TLR and STING agonists, may enhance future vaccination safety and efficacy [82]. New approaches are currently under scrutiny to increase antigen immune response and specificity.

## 6. Drug Delivery Systems (DDSs): Platforms, Formulations, and Clinical Profiles

DDSs are innovative technologies for the formulation and storage of drug molecules for targeted delivery [82,83,84]. In addition to transport, these systems can act as adjuvants, rapidly directing therapeutic agents to specific tissues to enhance efficacy and minimize side effects. By enabling precise drug release and superior pharmacological control, DDSs improve patient outcomes, and recent advances have emphasized the critical role of drug-release kinetics. Over the past few decades, DDSs have been developed for controlled and simplified release [83]. Each DDS has distinct physical, chemical, and morphological characteristics that influence its release rate and effectiveness [84,85]. The journey of controlled-release formulations began in the 1950s with sustained-release technology, allowing drug delivery for up to 12 h. By the 1980s, oral and transdermal formulations achieved therapeutic effects for up to 24 h. DDSs now offer advantages over conventional drugs, such as predetermined release rates and extended durations from days to years [86].

In 2020, a significant advancement in vaccine delivery was achieved with the introduction of LNP formulations for COVID-19 vaccines [87] and for cancer, H1N1, H3N2, and monkeypox virus vaccines in clinical trials. LNPs are leading non-viral vectors for delivering nucleic acids. They can efficiently encapsulate nucleic acids, protect them from degradation, enhance cellular uptake, and promote endosome escape, resulting in high transfection efficiency. LNPs can be formed with different lipid matrices depending on the target. They can be efficiently used for CRISPR-related gene editing [88]. The primary challenge now is developing practical, safe delivery platforms. Future advancements will hinge on innovations in materials science, nanotechnology, and bioengineering to improve antigen presentation and immune responses.

### Detailed Architecture of Lipid Nanoparticles (LNPs)

LNPs are the leading delivery system for mRNA therapeutics, especially demonstrated during the COVID-19 pandemic. Unlike traditional liposomes, LNPs have a core-shell structure that protects mRNA in a lipid matrix. Their performance relies on the synergistic interaction of four primary lipid components, formulated at specific molar ratios to optimize stability and transfection efficiency [88,89].

Over the past several decades, DDSs have undergone a transformative evolution to achieve precise, controlled, and simplified drug release. This journey began in the 1950s with the introduction of sustained-release technologies, such as the Spansule capsule, which extended therapeutic delivery for up to 12 h. By the 1980s, the development of sophisticated oral and transdermal formulations further pushed these boundaries, achieving consistent therapeutic effects for up to 24 h. Today, DDSs represent a cornerstone of modern medicine, offering advantages such as predetermined release rates and extended durations ranging from days to several years [90,91,92].

Among these advancements, LNPs have emerged as a breakthrough platform, particularly for their ability to be engineered with diverse lipid matrices tailored to specific biological targets, including efficient CRISPR-related gene editing [93,94,95]. The architecture of these LNPs is meticulously designed: helper lipids, such as 1,2-distearoyl-sn-glycero-3-phosphocholine (DSPC) or 1,2-dioleoyl-sn-glycero-3-phosphoethanolamine (DOPE), typically constitute about 10 mol% of the formulation. While DSPC provides essential structural stability to the LNP surface, DOPE is often preferred for its fusogenic properties; it can transition into an inverted hexagonal (HII) phase at low pH, directly promoting endosomal membrane fusion and enhancing the delivery of mRNA into the cytoplasm [94,95,96].

Sterols, primarily cholesterol, occupy a significant portion of the LNP structure (38.5–42.7 mol%), filling gaps between phospholipids to regulate membrane fluidity and ensure structural integrity during storage and transport. Recent research has also explored cholesterol analogs, such as β-sitosterol, to modify the particle’s internal crystalline structure and further boost delivery efficiency. Finally, PEGylated lipids are incorporated at low concentrations (~1.5 mol%) to act as surface stabilizers. These lipids create a hydration layer that prevents particle aggregation and inhibits the adsorption of serum proteins (opsonization), thereby extending the circulation half-life by delaying clearance by the mononuclear phagocyte system. There is a significant clinical trade-off: while PEG offers stability, high concentrations may hinder cellular uptake and have been associated with the production of anti-PEG antibodies, potentially reducing the effectiveness of subsequent doses [97,98].

## 7. Types of DDSs

### 7.1. Liposomes

**Figure 5:**
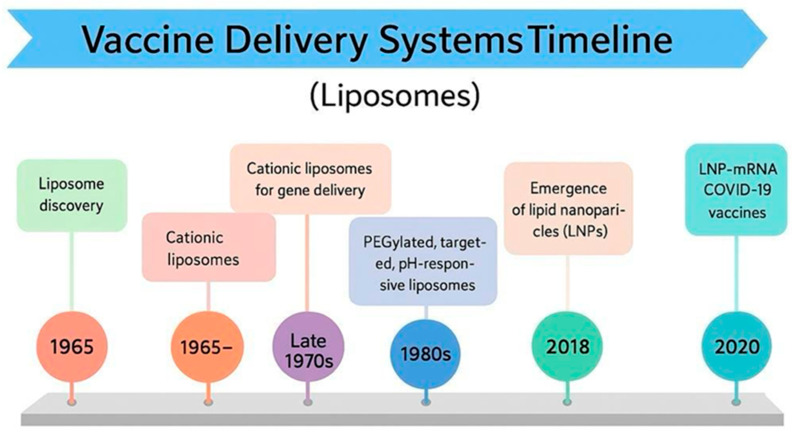
Historical evolution of lipid-based vaccine delivery systems (1965–2020). This timeline traces the progression from the foundational discovery of liposomes to modern mRNA-LNP technology. It highlights key shifts, including the development of gene delivery in the 1970s, the introduction of PEGylated and pH-responsive systems in the 1980s, and the development of specialized LNPs in 2018. This image was generated using Gemini AI.

Liposomes are self-assembled phospholipid vesicles used for drug delivery and can encapsulate both hydrophilic and lipophilic molecules. Their glycerophospholipid bilayer, stabilized by cholesterol, protects drugs from degradation and enables controlled release. In recent decades, liposomes have evolved from lab curiosities to clinically approved products, offering benefits like improved site targeting through the enhanced permeability retention (EPR) effect and better therapeutic outcomes with less toxicity than traditional formulations. Figure 5 illustrates the historical background of liposomes [99,100,101].

Next-generation delivery systems are integrating nanotechnology with new administration routes. Polymersomes are synthetic polymer vesicles with a hollow aqueous core and a bilayer of amphiphilic block copolymers [102]. Unlike liposomes composed of natural lipids, polymersomes offer improved mechanical stability, greater chemical diversity, and tunable membrane properties, including selective permeability. Polymersomes are more robust than lipid membranes due to their higher molecular weight and entangled structure, which prevents fusion and extends shelf life [102,103,104]. They allow extensive chemical modifications while maintaining self-assembly, making them ideal for advanced biomedical applications like drug delivery, gene therapy, and medical imaging [102,103,104]. Polymersomes can carry a range of therapeutics, including drugs and nucleic acids, and offer broader potential than liposomes. These innovative vaccine platforms are designed to address challenges such as cold-chain logistics and resource constraints in resource-limited countries [102,103,104].

### 7.2. Nanoparticle-Based Systems

Nanoparticle-based vaccine systems are a cornerstone of modern vaccinology, serving as innovative platforms that meticulously mimic viral structures and architectures to optimize antigen delivery and processing. Unlike vaccines and conventional vaccine formulations, these nanoparticles allow for self-assembled nanostructures that enable multivalent antigen presentation, which is essential for inducing B-cell receptor (BCR) clustering and robust primary activation. Furthermore, their high surface-to-volume ratio enables precise cargo loading and controlled release kinetics, thereby enhancing the stability of the molecular payload against extracellular enzymatic degradation [105,106,107,108] and enabling the engineering of the physicochemical properties of these carriers, such as size, zeta potential, and surface functionalization. It is possible to orchestrate targeted delivery to APCs, particularly dendritic cells. This targeted approach facilitates the efficient transport of antigens to secondary lymphoid organs, thereby amplifying immunogenicity and orchestrating the adaptive immune response [106,107,108].

**Figure 6:**
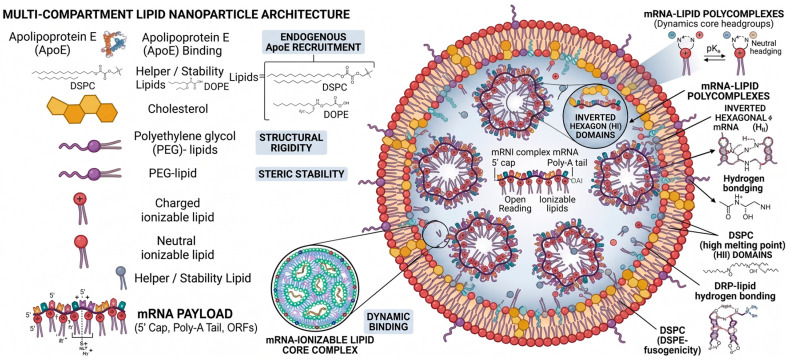
Structural Architecture and Molecular Composition of Multi-Compartment Lipid Nanoparticles (LNPs). This diagram illustrates the sophisticated, multi-compartment LNP architecture engineered for precise mRNA delivery. The internal core sequesters the mRNA payload stabilized by its 5′ cap, Poly(A) tail, and Open Reading Frames (ORFs)—within inverted hexagonal (H_II_) domains and dense core complexes to ensure structural rigidity. Stabilization of the genetic cargo is further enhanced by Degradable-Reduced-Polymer Lipids (DRP-lipids) through specialized hydrogen bonding. The particle’s biological interface is governed by ionizable lipids that undergo pH-dependent transitions to facilitate endosomal escape, while external PEG-lipids provide steric stability against immune clearance. Strategic incorporation of helper lipids, including 1,2-distearoyl-sn-glycero-3-phosphocholine (DSPC) and 1,2-dioleoyl-sn-glycero-3-phosphoethanolamine (DOPE), modulates bilayer fluidity. Furthermore, the recruitment of Apolipoprotein E (ApoE) facilitates receptor-mediated cellular uptake, while 1,2-distearoyl-sn-glycero-3-phosphoethanolamine (DSPE) enhances the particle’s fusogenicity and overall kinetic stability during therapeutic transport. This image was generated using Gemini AI.

A pinnacle of this bioengineering is the sophisticated, multi-compartment lipid nanoparticle (LNP) architecture, which has revolutionized the delivery of nucleoside-modified mRNA (Figure 6). This system operates as a dynamic, bio-responsive entity rather than a static carrier. At its core, the mRNA payload—stabilized by a 5′ cap, a Poly(A) tail, and strategically designed Open Reading Frames (ORFs)—is sequestered within a highly organized internal matrix. This core is characterized by inverted hexagonal H_II_ domains and dense core complexes that provide the necessary structural rigidity and facilitate the transition from a lamellar to a fusogenic state. The functional versatility of the LNP is primarily governed by ionizable lipids, which possess a specific acid dissociation constant (pKa) typically below 7.0. This allows the particles to maintain a near-neutral surface charge at physiological pH, minimizing non-specific interactions and systemic toxicity. Upon internalization via receptor-mediated endocytosis, the acidic environment of the maturing endosome triggers the protonation of these ionizable lipids. This transition induces a synergistic interaction with the anionic lipids of the endosomal membrane, leading to membrane destabilization and the subsequent “endosomal escape.” This process is vital for releasing the genetic payload into the cytosol before it can be trafficked to lysosomes for degradation. The external interface of the LNP is further refined by PEGylated lipids, which provide a steric barrier that prevents particle aggregation and reduces opsonization by plasma proteins. To ensure structural integrity and efficient delivery, specific helper lipids are strategically incorporated into the formulation. Saturated lipids such as DSPC and DSPE provide structural stability to the bilayer and often serve as anchors for PEG chains. Additionally, DOPE (1,2-dioleoyl-sn-glycero-3-phosphoethanolamine) enhances the particle’s fusogenic potential, while cholesterol is included to modulate bilayer fluidity and particle persistence [105,106,107,108].

This intricate interplay of molecular components is the fundamental driver of efficient cytosolic delivery. This process is paramount for high-fidelity antigen translation, which ultimately promotes potent CD8+ T cell activation and the production of high-affinity neutralizing antibodies. In the burgeoning field of cancer immunotherapy, researchers are leveraging these precision nanovaccines to reprogram the immunosuppressive TME via this same endosomal bypass, minimizing systemic cytotoxicity. Beyond efficacy, current research prioritizes thermostability challenges, exploring lyophilization techniques and the use of biomimetic, stimuli-responsive polymers to bypass stringent cold-chain requirements [109,110]. Furthermore, the horizon of vaccine administration is shifting toward minimally invasive routes, such as biodegradable microneedle patches and mucoadhesive nanocarriers for intranasal delivery. These innovations are designed to trigger a robust secretory IgA response, creating a first line of defense that prevents viral replication at the primary mucosal entry sites [111].

Innovative vaccine platforms, including polymersomes, microneedle patches, and intranasal sprays, aim to enhance systemic and mucosal immunity while reducing reliance on traditional injections and overcoming logistics constraints in resource-limited settings [112,113,114]. The convergence of nanotechnology and synthetic biology will inevitably lead to a new generation of single-dose vaccines characterized by unparalleled delivery efficiency and superior safety profiles.

## 8. Key Industrial Applications of Nanotechnology

Nanotechnology’s versatility is evident in its applications, especially in nanomedicine. It enhances disease treatment by using DDSs that target specific cells with nanoparticles, thereby improving efficacy and reducing side effects [114]. Additionally, nanoparticles serve as contrast agents in medical imaging (e.g., iron oxides for MRI and silica for CT), offering advantages over traditional radiation-based methods [115].

### 8.1. Fundamental Physicochemical Properties of Nanoparticles

Nanoparticles have unique properties that enhance their reactivity in biological environments, making them crucial for nanomedicine. Their small size (1–100 nm) offers a high surface-to-volume ratio, allowing easy infiltration into tissues and fluids [116,117,118,119]. Size affects key processes, including endocytosis and the ability to cross biological barriers, such as the blood-brain barrier; particles smaller than 200 nm enter cells via clathrin-coated vesicles.

Surface chemistry plays a crucial role in determining reactivity and function. Modifying surfaces, such as coating AuNPs and DNA with lipid layers, increases permeability and cellular uptake [120]. PEGylation of liposomes enhances bioavailability by reducing immune recognition, while ligand conjugation (e.g., folate or monoclonal antibodies) facilitates the selective targeting of cells, particularly cancer cells [121]. Additionally, surface chemistry enables environment-activated drug release in response to factors such as the acidic pH of tumors [122]. The shape of a nanoparticle is vital for its biological function. Spherical nanoparticles are generally more easily endocytosed than rod or tube-shaped ones due to their interaction with the cell membrane [123]. However, long nanorods can offer prolonged bioavailability and greater encapsulation capacity. Shape also influences the endocytosis pathway (clathrin-mediated vs. clathrin-independent) [124]. This tunability of physicochemical properties suggests that the future of nanomedicine is geared toward precision design, in which nanoparticles are tailored to specific therapeutic challenges [122,123,124].

### 8.2. Lipid-Based Carriers

Lipid-based carriers include micelles (5–50 nm) for hydrophobic drugs and liposomes (10 nm to several microns) that encapsulate both hydrophobic and hydrophilic drugs [125,126]. Liposomes play a crucial role in cancer treatment through the “enhanced permeability and retention effect” (EPR), which enables selective accumulation [127,128,129,130,131]. PEGylation extends half-life, while ligand conjugation facilitates active targeting. LNPs are also effective for nucleic acid therapies, such as CRISPR-Cas9 [132,133,134,135].

### 8.3. Dendrimers

Silica, magnetic, and gold nanoparticles play crucial roles in drug delivery. Dendrimers facilitate controlled drug release, while silica and magnetic nanoparticles enable targeted delivery [136,137,138]. Gold nanoparticles are functional for imaging and photothermal therapy [139,140,141]. The emergence of innovative drug delivery systems that respond to stimuli enhances control over drug release and localization. Nanocarrier encapsulation reduces the systemic toxicity of cytotoxic drugs like [142,143]. For instance, liposomal AgNP incorporation minimizes inflammation and enhances cytotoxicity at lower concentrations, providing a model for reducing drug toxicity [144].

## 9. Nanoparticle Cytotoxicity and Safety Considerations

The widespread use of nanotechnology has raised concerns about health and the environment, leading to the emergence of nanotoxicology [145,146,147]. The properties that enhance the pharmacological benefits of nanoparticles can also contribute to their toxicity, highlighting a trade-off between efficacy and safety, as optimizing cellular uptake and reactivity may increase the risk of unintended cellular damage [145,146,147,148]. Cytotoxic mechanisms involve tissue infiltration and disruption of cellular functions, primarily through the rupture of subcellular membranes and excessive production of reactive oxygen species (ROS), which leads to oxidative stress, DNA damage, and cell death [149,150]. Small nanoparticles show greater cytotoxicity due to enhanced cellular entry [151]. Surface modifications impact toxicity: charged nanoparticles are often more toxic, while PEGylation reduces uptake [152,153]. However, recent studies suggest that lipid PEGylation may enhance lipid nanoparticle delivery [154,155], opening new options for targeting specific tissues and cells.

## 10. Exosomes

Exosomes are vesicle-like structures, first identified in the 1980s, with diameters ranging from 40 to 1000 nm, which facilitate intercellular communication by transferring substances such as proteins, mRNAs, miRNAs, and lipids between cells [156,157]. Due to their greater biocompatibility and lower toxicity, they are being explored for drug-delivery applications; furthermore, exosomes secreted by immune cells can either stimulate or inhibit the immune response, with dendritic cell exosomes being explored for cancer and infectious disease vaccines [158,159].

Exosomes can enhance Th1-type responses and cell-mediated immunity, which is crucial for fighting viral and bacterial infections, and research specifically shows that exosomes with mycobacterial antigens induce a more effective Th1 response against *M. tuberculosis* in mice than protein subunit vaccines, which tend to promote Th2 responses and antibody-mediated immunity [126,160,161].

The synthesized evidence positions exosomes not merely as inert delivery vehicles, but as potent biological adjuvants that represent the next generation of rational vaccine design [162]. Their role as immunological helpers is fundamental to overcoming the limitations of traditional vaccinology. Unlike conventional aluminum salts (Alum), which are restricted to Th2-dominant humoral responses and often fail against intracellular pathogens, exosomes actively polarize the immune system toward Th1 and Th17 responses and robust CTL activity [163,164]. This intrinsic adjuvanticity is driven by a sophisticated molecular synergy: exosomes simultaneously deliver high-fidelity antigens and “danger signals,” including heat shock proteins (HSP70, HSP90) and specific lipids (oxidized phospholipids, lipid aldehydes, non-lysosomal sphingolipids, and acyl ceramides), which can activate the TLR4 and NLRP3 inflammasome pathways [165]. The efficiency of this biological adjuvancy is exemplified by “dose-sparing” effects, in which nanogram-level doses delivered via exosomes elicit immunity comparable to that elicited by microgram doses of protein subunit vaccines supplemented with chemical enhancers [166].

Furthermore, the transition from empirical to rational adjuvant design is facilitated by exosome engineering [167]. Platforms like StealthX™ prove that multivalent “mix-and-match” strategies can provide broad protection against evolving variants without the reactogenicity or “liver trap” effects associated with synthetic lipid nanoparticles [168,169]. While the chemistry, manufacturing, and controls bottleneck regarding yield and purification remains a critical hurdle for large-scale clinical implementation, the ability of exosomes to act as independent, biocompatible, and self-adjuvanted systems suggests they will redefine the standards of efficacy for both infectious disease prophylaxis and personalized cancer immunotherapy [170,171]. As regulatory frameworks evolve [171], the strategic integration of exosomes as primary helpers will likely be the cornerstone of non-invasive, needle-free, and high-precision global immunization efforts [172,173].

## 11. Induction of Tolerogenic Responses (Vaccines and Nanoparticles)

Tolerogenic vaccines represent a therapeutic strategy aimed at reinstating immune tolerance in the treatment of autoimmune disorders and allergic reactions [174,175,176]. This approach specifically targets and suppresses pathogenic immune responses associated with particular antigens, rather than activating them. In contrast to conventional vaccines, tolerogenic vaccines stimulate regulatory T (Treg) and B cells to mitigate autoimmunity without imposing broad immunosuppression. They demonstrate considerable potential to address conditions such as Type 1 Diabetes and allergic diseases [174,175,176]. Furthermore, transdermal tolerogenic nanoparticles have been used as an effective delivery system in asthma [177].

Further preclinical and clinical research is required in this domain, as the therapeutic effects of vaccines have not been adequately investigated.

## 12. Conclusions

The landscape of biological sciences is undergoing a fundamental transformation, marking a decisive shift from traditional, empirical vaccine design toward an era of molecularly programmed immunity. We have moved beyond simply boosting the immune system with basic aids; researchers are now leveraging synthetic biology and nanotechnology to engineer every functional component of the immune response precisely. The clinical success of advanced adjuvant systems such as AS01 and Matrix-M™ represents a critical milestone in overcoming the immunogenicity challenges posed by complex pathogens, including those responsible for malaria and RSV, that have historically evaded intervention. These achievements demonstrate that directed molecular synergy, achieved through the strategic engagement of multiple PRRs, is the most effective approach for eliciting the potent, targeted immune profiles required to address modern global health threats.

Looking toward the future, the distinction between “algorithm” and “adjuvant” is increasingly merging into a singular, optimized therapeutic entity. The integration of artificial intelligence frameworks, such as AlphaFold 3, now enables the prediction of T-cell recognition and MHC-peptide interactions with exceptional molecular accuracy, allowing for the rapid design of next-generation vaccine candidates. This computational revolution, combined with tools like CRISPR-Cas9 for scalable mRNA design, paves the way for “one-and-done” genetic treatments and chronic mRNA therapies for cancer and autoimmune diseases that were previously considered unattainable. Simultaneously, innovations in delivery vehicles, such as LNPs, are prioritizing endosomal escape and the use of next-generation selective organ target (SORT) lipids and poly-sarcosine to overcome the immunogenic limitations and biodistribution challenges associated with conventional PEGylation.

The next frontier of rational vaccine design centers on exosomes and biomimetic vesicles, which function not merely as inert carriers but as potent biological adjuvants. Their intrinsic ability to deliver high-fidelity antigens alongside biological “danger signals”—such as heat shock proteins and specific lipids—allows them to actively polarize the immune system toward Th1 and Th17 responses while maintaining a superior safety profile. Furthermore, the global future of vaccine administration lies in overcoming the “cold-chain bottleneck” through the vitrification of nanocomposites and the deployment of needle-free platforms, such as thermostable microneedle patches and mucoadhesive nanocarriers for intranasal delivery. These systems are specifically designed to induce potent mucosal IgA responses, blocking viral replication at the primary site of entry—a critical requirement for preventing future pandemics.

The 100-Day Mission to mitigate risks associated with the emergence of “Disease X” is an ambitious endeavor now feasible with current technological capabilities. This objective necessitates a profound shift away from outdated reliance on animal models toward a human-centric approach that leverages organ-on-a-chip technologies and AI-driven screening methods. However, the true significance of these technological milestones resides not in their technical complexity but in their ethical implications. Precision immunology must be inextricably linked with the moral imperative of global equity to ensure that health security is a universal biological certainty. Our final objective is to realize a future in which the highest caliber of scientific advancement safeguards every individual, regardless of their geographical location or economic status.

Ultimately, vaccines and efficient delivery systems have the potential to elicit tolerogenic responses, which may play a crucial role in the management of autoimmune and allergic diseases, particularly when pharmacological treatments are constrained.

## Data Availability

No new data were created or analyzed in this study. Data sharing is not applicable to this article.

## Glossary

DDS	Drug Delivery System
CTL	Cytotoxic T lymphocyte
DC	Dendritic Cells
TLR	Toll-like receptors
TLRL	Toll-like receptor ligands
AI	Artificial intelligence
PRRs	Pattern recognition receptors
Th1	T lymphocyte helper 1
Th2	T lymphocyte helper 2
Th17	T lymphocyte helper 17
dsRNA	DNA-capped double-stranded RNA
ARNAX	A synthetic DNA-capped double-stranded RNA (dsRNA) molecule used as a ligand of TLR3
CpG ODNs	Short, synthetic single-stranded DNA molecules containing unmethylated cytosine-guanine (CpG) dinucleotides
STING	Stimulator of IFN genes
Poly(I:C)	Polyinosinic: polycytidylic acid, activator of TLR3
LNP	Lipid nanoparticle
Cross-Reactive Material 197 (CRM197)	A non-toxic mutant of diphtheria toxin
MHC	Major Histocompatibility Complex
APC	Antigen-presenting cells
PAMP	Pathogen-Associated Molecular Patterns
DAMP	Damage-associated molecular patterns or alarmins
LPS	Lipopolysaccharide
HSP	Heat shock protein
HMGB1	High Mobility Group Box 1
MPL	Monophosphoryl lipid A
HPV	Human Papillomavirus
NLRP3	Nucleotide-binding domain, leucine-rich-containing family, pyrin domain-containing-3
TRIF	TIR-domain-containing adapter-inducing interferon β
MyD88	Myeloid Differentiation Primary Response 88
IRF	Interferon Regulatory Factors
LDHs	Layered Double Hydroxides
TME	Tumor Microenvironment
LRTD	Severe lower respiratory tract disease
ISCOMs	Immunostimulatory complexes
AS	Adjuvant Systems
DSPC	1,2-Distearoyl-sn-glycero-3-phosphocholine
DOPE	1,2-dioleoyl-sn-glycero-3-phosphoethanolamine
EPR	Enhanced permeability retention
PEG	Polyethylene glycol
BCR	B-cell receptor
ORFs	Open Reading Frames
AuNP	Gold nanoparticles
AgNP	Silver nanoparticles
ROS	Reactive oxygen species
T reg	T regulatory cells
SORT	Selective ORgan Targeting
